# A retrospective multicenter study of carbon‐ion radiotherapy for external auditory canal and middle ear carcinomas

**DOI:** 10.1002/cam4.1830

**Published:** 2018-12-08

**Authors:** Kazuhiko Hayashi, Masashi Koto, Yusuke Demizu, Jun‐ichi Saitoh, Hiroaki Suefuji, Tomoaki Okimoto, Tatsuya Ohno, Yoshiyuki Shioyama, Ryo Takagi, Hiroaki Ikawa, Kenji Nemoto, Takashi Nakano, Tadashi Kamada

**Affiliations:** ^1^ Hospital of the National Institute of Radiological Sciences National Institutes for Quantum and Radiological Sciences and Technology Chiba Japan; ^2^ Department of Radiology Hyogo Ion Beam Medical Center Tatsuno Japan; ^3^ Department of Radiology University of Toyama Toyama Japan; ^4^ Ion Beam Therapy Center SAGA‐HIMAT Foundation Tosu Japan; ^5^ Medicine & Biology Division Gunma University Heavy Ion Medical Center Maebashi Japan; ^6^ Department of Oral Surgery Tokyo Dental College Suidobashi Hospital Tokyo Japan; ^7^ Department of Radiation Oncology, Faculty of Medicine Yamagata University Yamagata Japan

**Keywords:** adenoid cystic carcinoma, carbon‐ion radiotherapy, carcinoma, external auditory canal, middle ear

## Abstract

**Background:**

We conducted a retrospective multicenter study to assess the clinical outcomes of carbon‐ion radiotherapy (CIRT) for head and neck malignancies (Japan Carbon‐Ion Radiation Oncology Study Group [J‐CROS] study: 1402 HN). We aimed to evaluate the safety and efficacy of CIRT in patients with external auditory canal (EAC) and middle ear (ME) carcinomas.

**Methods:**

Thirty‐one patients treated with CIRT at four Japanese institutions were analyzed. Fourteen patients (45.2%) had squamous cell carcinomas, 13 (41.9%) had adenoid cystic carcinomas, and four (12.9%) had other types. Nineteen (61.3%), six (19.4%), three (9.7%), and three (9.7%) patients had T4, T3, T2, and T1 disease, respectively. All patients had N0M0 status. The median radiation dose was 64 Gy (relative biological effectiveness) in 16 fractions. The median gross tumor volume was 33.3 mL.

**Results:**

The median follow‐up period was 18.4 months (range, 5.1‐85.6). The 1‐ and 3‐year local control and overall survival rates were 75.0% and 55.0% and 79.3% and 58.7%, respectively. Regarding grade 3 or higher toxicities, three patients (9.7%) had grade 3 dermatitis, one (3.2%) had grade 3 mucositis, and two (6.5%) had grade 3 central nervous necrosis (ie, radiation‐induced brain necrosis). No grade 4 or worse reactions were observed.

**Conclusion:**

CIRT was effective for EAC and ME carcinomas.

## 1 INTRODUCTION

Primary external auditory canal (EAC) and middle ear (ME) carcinomas are relatively rare diseases, with an incidence of one to six patients per 1 000 000 persons.^1^, ^2^ EAC and ME carcinomas are mainly treated with surgery. When the patient is diagnosed with locally advanced disease, postoperative radiation is performed.^3^ For inoperable cases or patients who refuse surgery, definitive radiotherapy is sometimes used. Ogawa et al reported that the disease‐free survival rate for patients with early‐stage disease was approximately 80% at 5 years after definitive radiotherapy, while the rates for patients with T2 and T3 disease according to the Stell classification were 45% and 0%, respectively.^3^ Additionally, Takenaka et al performed meta‐analyses of definitive chemoradiotherapy for locally advanced EAC carcinomas, which demonstrated that the 5‐year overall survival (OS) rate was 43.6%.^4^ Therefore, further research is expected to focus on attempts to improve the survival of patients with locally advanced EAC and ME carcinomas.

Carbon‐ion radiotherapy (CIRT) is a type of high linear energy transfer radiotherapy. CIRT demonstrates better dose‐localizing properties than photons^5^ and may improve the treatment outcome for EAC and ME carcinomas. To date, several promising results of CIRT for head and neck cancers have been reported by a single institute .^6^, ^7^, ^8^ The local control rate of EAC and ME carcinomas treated with CIRT was 54% after 3 years .^6^

To assess the clinical outcomes of CIRT for head and neck malignancies, a multicenter study was retrospectively conducted by the Japan Carbon‐Ion Radiation Oncology Study Group (J‐CROS) (J‐CROS 1402 HN). The clinical outcomes for each major histological type have already been reported, and CIRT demonstrated promising results.^9^, ^10^, ^11^, ^12^ To elucidate the safety of CIRT, sub‐analyses based on primary tumor sites may be useful in clinical situations. In this study, we evaluated the clinical outcomes of patients with EAC and ME carcinomas using data from J‐CROS 1402 HN.

## 2 PATIENTS AND METHODS

### 2.1 J‐CROS 1402 HN

J‐CROS 1402 HN was conducted as a retrospective survey of patients with a primary or recurrent head and neck malignancy who received CIRT in four institutions in Japan between November 2003 and December 2014. The inclusion criteria were as follows: (a) histologically confirmed malignancy, (b) no bone or soft tissue tumors, (c) N0 or N1M0 status, (d) medically inoperable tumors or surgery refusal, (e) definitive intent, (f) measurable tumors, and (g) an Eastern Cooperative Oncology Group performance status of 0‐2. Patients who had previously undergone irradiation for the same lesion were excluded.^9^ This study was conducted according to the guidelines approved by the Institutional Review Board of each institution and was registered with UMIN‐CTR (www.umin.ac.jp/ctr/index-j.htm) (identification number UMIN000024473).

The survey included 908 eligible patients. Of these, 31 patients with EAC and ME carcinomas were enrolled in this study. Primary tumors were classified according to the Pittsburgh Staging System.^13^ Lymph node status was classified using the 7th edition of the TNM staging system for cancers of the head and neck region. Local control was defined as no evidence of tumor regrowth in the planning target volume (PTV). Regional control was defined as no evidence of recurrence in the regional lymph nodes and temporal bone beyond PTV.

The National Cancer Institute's Common Terminology Criteria for Adverse Events version 4.0 was the preferred method for determining toxicities after treatments .^14^ Acute toxicity was defined as that occurring within 3 months from the initiation of CIRT, while late toxicity was defined as that occurring >3 months after initiation of CIRT. We collected information on grade 3 or higher acute toxicities and grade 2 or higher late toxicities.

### 2.2 Carbon‐ion radiotherapy

Patients were positioned in customized cradles and immobilized using a low‐temperature thermoplastic shell. Computed tomography (CT) images of all patients fixed in position using an individually tailored immobilization device were taken in the supine position. Using the CT images, a 3‐dimensional treatment plan was designed. We contoured the gross tumor volume (GTV) on the CT images using magnetic resonance imaging scans as reference. The clinical target volume (CTV) included the GTV with a minimum added margin of 5 mm. The PTV included the CTV with added margins of 2‐5 mm.

The dose was prescribed to the isocenter. The PTV was conformally enclosed at a minimum of 90% isodose line with the prescribed dose. Irradiation was almost always performed in 3‐6 fields with carbon‐ion beams. Based on this trial, the limiting doses for critical normal tissues were also defined, with a maximum point dose of 30 Gy (RBE) allowed for the brain stem. However, one institution adopted a CIRT schedule with 26 or 32 fractions and used a dose constraint of 48 Gy (RBE) for the brain stem.

The most common prescribed dose was 64 Gy (RBE) in 16 fractions (14 patients, 45.2%), followed by 57.6 Gy (RBE) in 16 fractions (seven patients, 22.6%) and 65.0 Gy (RBE) in 26 fractions (six patients, 19.4%; Table 1).

**Table 1:** Patient and tumor characteristics

Factors	Value or number (%)
Sex	
Male	13 (41.9)
Female	18 (58.1)
Age, years	
Median/range	55/29‐79
PS	
0	16 (51.6)
1	13 (41.9)
2	2 (6.5)
Disease status	
Initial disease	23 (74.2)
Recurrent disease	8 (25.8)
Histology	
Squamous cell carcinoma	14 (45.2)
Adenoid cystic carcinoma	13 (41.9)
Adenocarcinoma, not other specified	2 (6.5)
Others	2 (6.5)
Operability	
Yes	17 (54.8)
No	14 (45.2)
Chemotherapy	
Yes	5 (16.1)
No	26 (83.9)
Clinical T classification	
1	3 (9.7)
2	3 (9.7)
3	6 (19.4)
4	19 (61.3)
Clinical N classification	
0	31 (100)
1	0 (0)
Prescribed dose (BED10)	
57.6 Gy (RBE)/16 fr (78.3 Gy (RBE)	7 (22.6)
60.8 Gy (RBE)/16 fr (83.9 Gy (RBE)	1 (3.2)
64.0 Gy (RBE)/16 fr (89.6 Gy (RBE)	14 (45.2)
65.0 Gy (RBE)/26 fr (81.3 Gy (RBE)	6 (19.4)
70.4 Gy (RBE)/32 fr (85.9 Gy (RBE)	3 (9.7)
GTV, mL	
Median/range	33.3/0.8‐271.3

BED, biologically effective dose; fr, fractions; GTV, gross tumor volume; PS, performance status; RBE, relative biological effectiveness.

The biologically effective dose (BED) was calculated on the basis of a linear‐quadratic model, assuming an α/β ratio of 10 for the tumor, to compare various fractionation doses.^15^ The dose of 64 Gy (RBE) in 16 fractions corresponded to BED10 = 89.6 Gy (RBE), which was the highest and most common dose used.

### 2.3 Statistical analysis

Local control, OS, and progression‐free survival (PFS) were calculated using the Kaplan–Meier method. All survival times were calculated from the first day of CIRT. Univariate analyses of prognostic factors for local control and OS were performed using the log‐rank test. Continuous characteristics such as age, BED, and GTV were divided into two subgroups using the median values. Statistical significance was set at *P* < 0.05. We used JMP statistical software version 13.0 (SAS Institute Inc, Cary, NC, USA) for all statistical analyses.

Fisher's exact tests were used to compare the incidence of late toxicities between patients receiving BED10 <89.6 Gy (RBE) and those receiving BED10 = 89.6 Gy (RBE).

## 3 RESULTS

### 3.1 Patient, tumor, and treatment characteristics

Thirty‐one patients with EAC and ME carcinomas were analyzed. Table 1 presents the patient and tumor characteristics. All patients had N0M0 status. Five patients received neoadjuvant chemotherapy with the following regimens: cisplatin (one patient), cisplatin+5‐fluorouracil (two patients), and tegafur/gimeracil/oteracil (two patients). No patient received concurrent or adjuvant chemotherapy.

**Figure 1:**
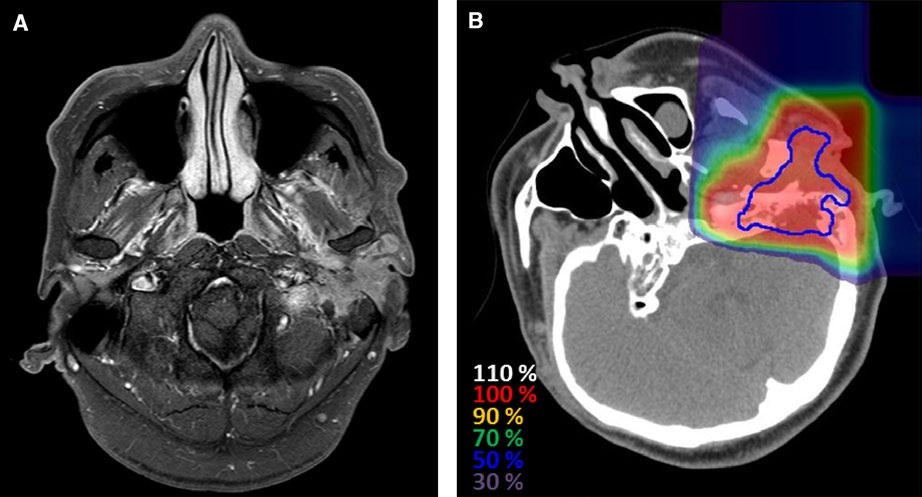
Representative case of a patient with squamous cell carcinoma of the left middle ear who was treated with carbon‐ion radiotherapy. A, Magnetic resonance imaging before carbon‐ion radiotherapy reveals a well‐enhanced left middle ear tumor B, Dose distribution. Blue lines show gross tumor volume

Figure 1 shows a representative case of a patient with left ME carcinoma who was treated with CIRT.

### 3.2 Local control and survival

**Figure 2:**
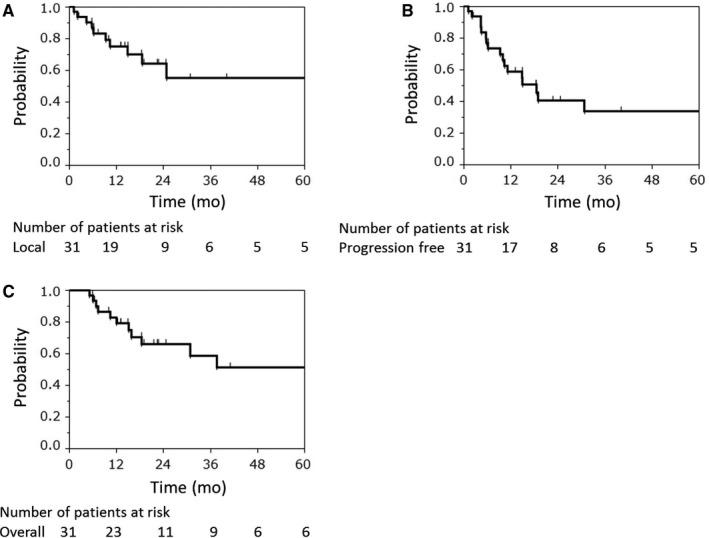
Local control rate (A), progression‐free survival rate (B), and overall survival rate (C) of all patients (n = 31)

The median follow‐up period of all 31 patients was 18.4 months (range, 5.1‐85.6). At the first recurrence site, nine patients had local recurrence. Of these, eight patients had local recurrence within PTV and one patient had recurrence within the margin of the PTV. Two patients had regional lymph node metastases, and seven had distant metastases. At the final observation, 12 patients died from their disease and none died from unrelated causes. The 1‐, 3‐, and 5‐year local control rates were 75.0% (95% confidence interval [CI], 55.8‐87.7), 55.0% (95% CI, 31.9‐76.1), and 55.0% (95% CI, 31.9‐76.1), respectively (Figure 2A). The 1‐, 3‐, and 5‐year PFS rates were 58.7% (95% CI, 40.3‐74.9), 33.7% (95% CI, 23.2‐60.5), and 33.7% (95% CI, 23.2‐60.5), respectively (Figure 2B). The 1‐, 3‐, and 5‐year OS rates were 79.3% (95% CI, 60.9‐90.4), 58.7% (95% CI, 37.2‐77.4), and 51.4% (95% CI, 29.7‐72.6), respectively (Figure 2C).

### 3.3 Toxicity

Regarding acute toxicities, three patients (10%) had grade 3 dermatitis and one (3%) had grade 3 mucositis (Table 2). Regarding late toxicities, two patients (6%) developed grade 3 central nervous system necrosis, that is, radiation‐induced brain necrosis. They were treated with steroid or surgical intervention, and consequently, their symptoms improved. There were three patients with grade 2 external ear inflammation (10%) and one patient with grade 2 tinnitus (1%). No grade 4 or worse toxicities were observed.

**Table 2:** Toxicity

Grade	2 (%)	3 (%)	4 (%)
Acute			
Mucositis		3 (9.7)	0
Dermatitis		1 (3.2)	0
Late			
Central nervous system necrosis	0	2 (6.5)	0
External ear inflammation	3 (9.7)	0	0
Tinnitus	1 (3.2)	0	0

In addition, we found that the incidence of grade ≥2 late toxicities was not significantly lower in patients who received BED10 <89.6 Gy (RBE) than in those who received BED10 = 89.6 Gy (RBE) (29.4% vs 7.1%, respectively; *P* = 0.185; Table S1).

### 3.4 Prognostic factors

Univariate analysis was performed to explore potential prognostic factors for local control and OS among subgroups (Table 3). These results did not identify any significant prognostic factors. However, the analysis of local control indicated that squamous cell carcinoma (SCC) was a nonsignificantly poorer prognostic factor in comparison with adenoid cystic carcinoma and other histological types (*P* = 0.055). Comparing adenoid cystic carcinomas and SCCs showed 3‐year local control rates of 76.2% (95% CI, 39.3‐94.1) and 30.3% (95% CI, 8.8‐66.3), respectively, 3‐year PFS rates of 45.1% (95% CI, 19.1‐74.1) and 32.1% (95% CI, 11.4‐63.4), respectively, and 3‐year OS rates of 83.1% (95% CI, 51.5‐95.8) and 45.4% (95% CI, 19.2‐74.4), respectively. Associations between GTVs and outcomes were also evaluated. Although no significant difference was identified, the 3‐year local control rates were 71.8% (95% CI, 44.1‐89.2) for GTVs <33.3 mL and 44.4% (95% CI, 17.3‐75.3) for GTVs ≥33.3 mL.

**Table 3:** Univariate analysis for local control and overall survival rates

Parameters	No. of patients	Local control	Overall survival
		*P*‐value	*P*‐value
Sex			
Male	13	0.875	0.222
Female	18		
Age			
<55 years old	14	0.964	0.333
≥55 years old	17		
Disease status			
Initial disease	23	0.762	0.627
Recurrent disease	8		
Histology			
SCC	14	0.055	0.256
ACC	13		
Others	4		
Operability			
Yes	17	0.499	0.627
No	14		
Clinical T classification			
1‐3	12	0.564	0.364
4	19		
BED			
<89.6 Gy (RBE)	17	0.121	0.311
=89.6 Gy (RBE)	14		
GTV			
<33.3 mL	15	0.211	0.355
≥33.3 mL	16		

ACC, Adenoid cystic carcinoma; BED, biologically effective dose; GTV, gross tumor volume; RBE, relative biological effectiveness; SCC, squamous cell carcinoma.

## 4 DISCUSSION

External auditory canal (EAC) and middle ear (ME) carcinomas are rare diseases and, to date, the use of CIRT for EAC and ME carcinomas has only been evaluated in a single study, which was based on single‐institution data 6. To the best of our knowledge, this is the first multi‐institutional study on CIRT for EAC and ME carcinomas. Our findings demonstrated that CIRT is effective for EAC and ME carcinomas, especially adenoid cystic carcinoma. Therefore, CIRT for EAC and ME carcinomas is a promising treatment option, especially in patients with inoperable locally advanced carcinomas.

For EAC and ME carcinomas, photon radiotherapy plays the role of adjuvant therapy because the first choice of treatment for such tumors is surgery; however, definitive photon radiotherapy for inoperable cases or for patients who refuse surgery has been reported in several studies.^3^, ^16^, ^17^ It is difficult to compare the studies directly because several staging systems of EAC and ME carcinomas have been used. Nonetheless, we have compared the studies in a general manner. Ogawa et al conducted a multicenter study using conventional photon radiotherapy for SCCs of the EAC and ME and reported that patients receiving definitive radiotherapy for T2 and T3 tumors (Stell classification) had 5‐year local control of 45% and 0%, respectively, as well as 5‐year disease‐free survival rates of 45% and 0%, respectively .^3^ Madsen et al performed a nationwide study in Denmark and reported that 26 patients with EAC and ME carcinomas were treated with radiotherapy alone. Although the details of clinical T classification were unclear and their study included SCCs (69%) and other histologies (31%), the 3‐year locoregional control and OS rates were 47.4% and 25.6%, respectively .^16^ Koto et al treated 13 patients with SCCs of the EAC and ME in the T3 or T4 stage, according to the modified Pittsburgh grading system of classification using carbon‐ion radiotherapy. They revealed that the 3‐year local control and OS rates were 54% and 40%, respectively .^6^ In comparison, our study included 10 patients with T3 or T4 SCCs and revealed that the 3‐year local control, PFS, and OS rates were 30.3%, 32.1%, and 45.4%, respectively. These rates were achieved using CIRT for advanced SCCs of the EAC and ME and may indicate comparable local control and survival outcomes to those obtained with photon radiotherapy.

Especially for cases of SCC, new strategies will be essential to achieve further improvements in treatment outcomes. Some previous studies have reported moderate efficacy associated with the use of concurrent photon radiotherapy plus chemotherapy, such as with platinum‐based drugs, 5‐fluorouracil, and docetaxel.^4^, ^18^ Extrapolating the results of these studies to CIRT suggests that outcomes might be improved by additional chemotherapy, for example, with platinum‐based drugs, 5‐fluorouracil, and docetaxel. Additionally, it is notable that nine patients in the current study had local recurrence at the first recurrence site, of whom eight had local recurrence within PTV. However, the incidence of grade ≥2 late toxicities was not significantly lower in patients who received BED10 <89.6 Gy (RBE) than in those who received BED10 = 89.6 Gy (RBE). Therefore, further dose escalation may be possible.

No study has been reported on photon or carbon‐ion radiotherapy for EAC and ME carcinomas, especially adenoid cystic carcinomas. However, Sulaiman et al conducted a multicenter study on adenoid cystic carcinoma of the head and neck treated with CIRT in 289 patients. The 2‐ and 5‐year local control rates were 88% and 68%, respectively,^12^ which is somewhat consistent with the local control rate of 76.2% at 3 years in our study.

Regarding the toxicities associated with radiotherapy, acute mucositis and dermatitis were controllable by conservative treatment and were improved after CIRT. In contrast, it is difficult to treat late toxicity; therefore, steps should be taken to avoid severe late toxicity. Ogawa et al illustrated that among 87 patients with EAC and ME carcinomas (including 53 patients treated with postoperative radiotherapy) who were treated with photon radiotherapy, grade 4 osteoradionecrosis with skin ulcers occurred in two patients.^3^ However, they reported that no grade 3 or 5 late toxicity was observed. Pemberton et al performed photon radiotherapy in 123 patients with EAC and ME carcinomas and demonstrated that bone and soft tissue necrosis occurred in six and two patients, respectively .^19^ In Koto et al’s study of 13 patients receiving CIRT for T3‐T4 SCC of the EAC and ME, six patients developed grade ≥2 late toxicities after CIRT, including two patients who had grade 3 temporal bone necrosis with skin ulcers, and four patients who had grade 2 brain necrosis .^6^ In the present study, two patients who received CIRT in the early days of our study had grade 3 late central nervous system necrosis. No patient developed grade 4 or higher late reactions. For central nervous system necrosis induced by CIRT, a previous study demonstrated that the brain volume receiving >50 Gy was a significant risk factor for the development of grade 2 or higher radiation‐induced brain injury .^20^ Future studies should focus on using new technical methods such as scanning irradiation or intensity‐modulated particle therapy, since they improve dose distribution 21, 22, 23 and may therefore reduce the incidence of central nervous system necrosis. Meanwhile, our results demonstrated that there was no grade 2 or higher osteoradionecrosis, although Koto et al reported grade 3 temporal bone necrosis with skin ulcers in two patients receiving CIRT .^6^ They also reported that the two patients with temporal bone necrosis were treated at the beginning of CIRT and their conditions exacerbated because of the skin ulcers. Skin dose was recently reduced to the least possible extent, and the irradiated fields were localized; therefore, we may be able to reduce the incidence of bone necrosis.

However, our study had some limitations. First, our study was conducted using retrospective data. Second, only 31 patients were included in our study as EAC and ME carcinomas are rare. Third, the median follow‐up period was short (18.4 months).

Our multicenter study (J‐CROS 1402 HN) showed that CIRT was effective and safe for head and neck malignancies, particularly including radioresistant tumors.^9^, ^10^, ^11^, ^12^, ^23^, ^24^, ^25^ Since April 2018, the public health insurance system in Japan has covered CIRT for head and neck malignancies, with the exception of radiosensitive oral, laryngeal, and pharyngeal squamous cell carcinoma.

In conclusion, our study demonstrated that definitive CIRT is effective for EAC and ME carcinomas, especially adenoid cystic carcinoma. In the future, multi‐institutional prospective studies with a large number of patients are warranted to further analyze the findings of our study.

## CONFLICT OF INTEREST

The authors declare that they have no conflicts of interests.
