# Supplementary material for: A retrospective multicenter study of carbon‐ion radiotherapy for external auditory canal and middle ear carcinomas
Source: Cancer Med. 2018 Dec 8;8(1):51–7. doi: 10.1002/cam4.1830 (PMC6346229; doi:10.1002/cam4.1830)
Supplement: Supplementary file 1 [file CAM4-8-51-s001.docx]

Table S1 Late toxicities and prescribed dose

|  | Grade 2  *n* (%) | Grade 3  *n* (%) |
| --- | --- | --- |
| Central nervous system necrosis  　　　 BED10 < 89.6 Gy (RBE)  BED10 = 89.6 Gy (RBE) | 0 | 2 (6.5)  1  1 |
| External ear inflammation  BED10 < 89.6 Gy (RBE)  BED10 = 89.6 Gy (RBE) | 3　(9.7)  3  0 | 0 |
| Tinnitus  BED10 < 89.6 Gy (RBE)  BED10 = 89.6 Gy (RBE) | 1 (3.2)  1  0 | 0 |

Abbreviations: BED, biologically effective dose; RBE, relative biological effectiveness
